# The repurposing of recreational drugs as medical treatments

**DOI:** 10.1017/S109285292510028X

**Published:** 2025-10-24

**Authors:** Seetal Dodd, Stephen M. Stahl, Michael Berk

**Affiliations:** 1 School of Medicine, Deakin University, Geelong, VIC, Australia; 2 Department of Psychiatry, The University of Melbourne, Melbourne, VIC, Australia; 3 Mental Health - Drug and Alcohol Services, University Hospital Geelong, Barwon Health, Geelong, VIC, Australia; 4 Neuroscience Education Institute, Cranbury, NJ, USA; 5 Department of Psychiatry, University of California San Diego, San Diego, CA, USA

**Keywords:** drug repurposing, criminalization, stigma, drug policy, traditional medicine, psychoactive drugs

Drugs that are used for purposes other than medical treatment have been a part of the sociocultural landscape since antiquity, and the crossing over of drugs between recreational use, ritual and cultural use, and medical treatment can also be demonstrated with examples throughout history. Many ancient and traditional belief systems perform rituals that include psychoactive drugs as an integral part of treatment. In modern times, pharmacotherapy has become increasingly organized and regulated, and recreational drugs criminalized and excluded from the clinical setting. A dichotomy has developed between recreational drugs and medical pharmacotherapies that is challenged by emerging evidence and clinical use.

The exclusion of drugs now labelled “recreational” or “illicit,” has a relatively short history. Early prohibition laws focused initially on alcohol and later on pharmaceuticals. These were reactive to a perceived social or moral problem. Cocaine and morphine were restricted in the USA from 1914, available only with a prescription. Criminalization of cannabis accelerated with the formation of the Federal Bureau of Narcotics in 1930 in the USA.

With criminalization, stigmatization and misinformation also became a feature of cultural attitudes toward non-prescription drug use, as well as the association of drug use with counterculture, jazz music, beat poets, and later non-conformist groups. Research and access became increasingly more restricted ending definitively with the introduction of the Controlled Substances Act in 1970 and coinciding with the “War on Drugs,” a global effort lead by the USA to eradicate illicit drugs using strategies such as greatly increased penalties, enforcement, and incarceration.

Recently, there has been an easing of some of the laws restricting recreational drug use and with these changes new opportunities have emerged for further research and treatment using drugs that were previously heavily restricted. Decriminalization of recreational drug use is an evolving reform that prevents drug users with no other criminal involvement from being caught up in the justice system. The relaxation of laws restricting recreational drugs has also created an environment where repurposing recreational drugs for medical use has become possible, with new legal frameworks, regulatory approvals, drug formulations, and guidelines, permitting previously illicit drugs to fit into a modern medical environment.

There are well-established pathways that the industry uses to develop a new pharmaceutical and bring it to market as a new treatment. Regulatory authorities expect processes to be followed and carefully check for compliance. Medical practitioners are reliant on established processes and the vigilance of regulatory authorities so that they can be confident that the treatments they are prescribing are evidence based, effective, and safe. Regulatory pathways also provide medical practitioners with important information to assist them with clinical decision making, such as indications, contra-indications, dosing recommendations, adverse effects, and drug interaction risks. The repurposing of recreational drugs as medical treatments does not follow conventional pathways for regulatory approval. There is a paucity of preclinical data and important clinical information for some drugs, and clinical trials are often conducted with small budgets and small sample sizes. Data quality often doesn’t reach the standard asked by regulatory authorities from the pharmaceutical industry when registering a new product.

Legalization of medicinal cannabis in California USA was achieved when Proposition 215, the Compassionate Use Act of 1996^1^ was voted into law, bypassing the usual processes of the US Food and Drug Administration. Legalization of psilocybin and MDMA for medical use in Australia in 2023 was achieved when the regulatory body reclassified the two substances from a schedule 9 to a schedule 8 listing, in response to over 6,000 public submissions and an expert report,^2^ even though their evidence and usefulness are still debated.^3^ Approval processes that are non-conventional may be subject to legal challenge, as was the case with Proposition 215 which was challenged in the US Supreme Court. Proposition 215 legalized medicinal cannabis without removing the restrictions on medical research using cannabis and without changing federal laws, with the Medical Marijuana Regulation and Safety Act passed in California in 2015.

In contrast, ketamine has a history of medical and veterinary use, which has facilitated its repurposing as an antidepressant. It differs from other agents, in that it has existing regulatory approval as an anesthetic agent, facilitating translation and implementation. Approval processes for each recreational drug repurposed for medical use are considered on a case-by-case basis and may vary between drugs.

Where a recreational drug is shown to be an effective treatment for a mental health indication, there are still considerations regarding dosing, formulation and safety that need to be addressed through research.

## Traditional and alternative treatments

Psychoactive drugs have been used by some indigenous communities in rituals and traditional medicine. Treatment has often had mystical or spiritual goals congruent with the belief systems of the indigenous groups. Use by alternative therapy practitioners may aim for mystical experiences as treatment goals or have made claims of benefits of treatment without providing evidence.

Arguably, to traditional indigenous groups and to alternative medicine practitioners, the need to demonstrate that treatments are efficacious for illnesses that are recognized by conventional medical practitioners may not be something that they consider necessary, or even of any value at all. Similarly, patients who engage in traditional or alternative therapies may be attracted to the spiritual or mystical goals of the practitioner. Balancing benefit with harm may be complicated.

## Role of activists

Activists have been prominent and their demands and legal maneuvres have often spearheaded changes. This is especially the case with medicinal cannabis, but also with psilocybin and MDMA, and will continue to be a prominent driver of further reforms. Activists have diverse objectives, backgrounds and motivations. Recreational drug users who are pursuant of wide reforms of drug laws may support medical repurposing as they can see that recreational drug users may benefit from these reforms. They may also genuinely believe that some recreational drugs have therapeutic value. Patients, especially those that have responded inadequately to conventional therapies, will often have a hopeful view toward controversial treatments and have been prominent activists for legal reforms. Patient advocacy and commercial interests have capacity to lobby politicians and engage lawyers.

Activist led reforms can be controversial. An example is the trojan horse strategy, where pressure for medicinal use of recreational drugs is used as a mechanism for furthering an agenda of recreational use. A medicinal marijuana card is sought by recreational marijuana users. The actions of activists have broken the barriers that limit access to recreational drugs, and they are sometimes willing to by-pass or challenge the regulatory system that oversees medical treatments. The US Food and Drug Administration has a mandate to regulate against products that make false therapeutic claims, a problem that was unregulated in the USA prior to Sherley Amendment of 1912.^3^ The medical establishment is resistant to efforts to soften regulatory requirements for new pharmaceuticals, mindful that most proposed new treatments either fail to show expected clinical benefits or demonstrate clinical risks. To the medical establishment, the laws that made recreational drug use illegal, and the regulations for the registration of pharmaceutical treatments, are very different things. To activists, both may be regarded as barriers.

## Mainstream views

Use of recreational drugs remains highly stigmatized in the general public, due to perceived harms and their association with sub-cultures and criminal activity. Yet elsewhere, media reports have referred to repurposed recreational drugs using terms such as “breakthroughs,” “wonder drugs,” and “miracle medicine.” In addition, patient acceptance of novel therapies may be influenced by the therapeutic alliance they have with their treating clinician. Diversity of views is considerable in the broader community and influences such as upbringing, views of peers, and personal experiences will also influence views held by individuals.

## Attitudes of researchers, academics, and clinicians

Health professionals and researchers are trained to be skeptical, approving new advances when convinced by the evidence. In theory, they are impartial if a new treatment has an illicit history and look only at the evidence. In practice, they are subject to the same influences as the broader community. They have diverse views on recreational drug repurposing, ranging from opposition to advocacy, with nuanced views being common. These views are facilitated by evidence that is not overwhelming, negative studies, adverse effects, and people holding supportive views for some repurposed drugs and opposing views for others. Clinical experience can be re-affirming, observing patients who respond that had previously been non-responsive to conventional treatments, or repudiating through non-response, adverse effects, or experience with prescription seeking recreational drug users. With the regulatory approval of some of these drugs, the views of key opinion leaders and unpublished clinical experience can be decisive in firming opinions. As is often the case in the public arena, the smaller number of voices at the extremes are generally louder than the large number in the center. Activist views from within the medical profession exist, as do concerns about the public health consequences of repealing laws that restrict access to recreational drugs. Conclusions about the clinical utility of a drug are reached by balancing the benefits of treatment with the risks, however, benefits and risks can be evaluated differently, especially when evidence is limited.

## References

[1] Marmor JB. Medical marijuana. West J Med. 1998;168(6):540–543. 9656007 PMC1305083

[2] Nogrady B. Australia’s approval of MDMA and psilocybin for PTSD and depression is premature, say critics. BMJ. 2023;382:1599 37433614 10.1136/bmj.p1599

[3] United States Supreme Court. The Sherley amendment to the pure food and drugs act is constitutional. A misbranded “patent medicine condemned” seven cases Eckman’s alterative v. United States, (1916). Public Health Rep (1896–1970) 1916;31(3):137–140. http://www.jstor.org/stable/4573181.

